# Antiviral effect of eosinophils in respiratory tract infection with influenza virus in mice

**DOI:** 10.1186/1476-9255-10-S1-P24

**Published:** 2013-08-14

**Authors:** Suzanne M Bal, Annemiek Dijkhuis, Koen van der Sluijs, Rene Lutter

**Affiliations:** 1 Department of Experimental Immunology, Academic Medical Centre, University of Amsterdam, Meibergdreef 9, 1105 AZ Amsterdam, The Netherlands; 2 Department of Respiratory Medicine, Academic Medical Centre, University of Amsterdam, Meibergdreef 9, 1105 AZ Amsterdam, The Netherlands; 3 Laboratory of Experimental Intensive Care and Anesthesiology, Academic Medical Centre, University of Amsterdam, Meibergdreef 9, 1105 AZ Amsterdam, The Netherlands

## Rationale

Previous studies in an acute house dust mite murine model of allergic asthma have shown that allergic mice have an improved antiviral response to infection with influenza (A/PR/8/34) compared to non-allergic mice as reflected by lower viral titres in the lung and decreased weight loss. These findings demonstrate that allergic mice are able to clear the infection faster. Interestingly, we observed a synergistically enhanced eosinophilic response in bronchoalveolar lavage (BAL) of these mice in response to the viral infection.

## Hypothesis

This led us to hypothesise that eosinophils are important for the antiviral response and we tested this hypothesis in IL-5 transgenic (IL-5 Tg) mice, which present with chronic eosinophilia.

## Methods

IL-5 Tg mice and wild type C57BL/6 were infected with 10 TCID_50_ influenza (A/PR/8/34). Plasma and BAL were collected on day 4, 6 or 8 after infection and BAL cells were analysed by flow cytometry. The viral load was determined in lung tissue by qPCR. Before infection, IL-5 Tg mice had plasma levels of 500 pg/ml IL-5 and elevated eosinophils in the circulation (40% of cells), the spleen (35%), the lungs (25%) and BAL (4%).

## Results

Upon influenza infection there was a significantly elevated influx of eosinophils into the lungs as measured in the BAL which peaked at day 4 after infection: IL-5 Tg animals had 6.9 * 10^4^ eosinophils (10%) compared to 0.6 * 10^4^ (2%) in wild type animals (p = 0.01). Equal numbers of neutrophils, macrophages and lymphocytes were found in both groups. In concurrence with our hypothesis the IL-5 Tg mice rapidly recovered from the infection. Their maximal weight loss was limited to 9%, compared to 22% in wild type animals and was paralleled by a significantly lower viral load in the Tg animals at day 8 after infection: log viral copies of 4.2 compared to 6.5 (p = 0.02).

## Conclusion

These data imply that eosinophils play a crucial role in the immune response against influenza virus in mice and underline the importance of the innate antiviral immunity. Whether this applies to humans and, if so, whether this is different for patients with asthma, remains to be addressed.

